# A Novel Rat Model of Type 2 Diabetes: The Zucker Fatty Diabetes Mellitus ZFDM Rat

**DOI:** 10.1155/2013/103731

**Published:** 2013-02-26

**Authors:** Norihide Yokoi, Masayuki Hoshino, Shihomi Hidaka, Eri Yoshida, Masayuki Beppu, Ritsuko Hoshikawa, Katsuko Sudo, Akihiko Kawada, Sadaaki Takagi, Susumu Seino

**Affiliations:** ^1^Division of Cellular and Molecular Medicine, Department of Physiology and Cell Biology, Kobe University Graduate School of Medicine, 7-5-1 Kusunoki-cho, Chuo-ku, Kobe 650-0017, Japan; ^2^Division of Molecular and Metabolic Medicine, Department of Physiology and Cell Biology, Kobe University Graduate School of Medicine, Kobe 650-0017, Japan; ^3^Hoshino Laboratory Animals, Inc., Ibaraki 306-0606, Japan; ^4^Animal Research Center, Tokyo Medical University, Tokyo 160-8402, Japan; ^5^Japan SLC, Inc., Hamamatsu 431-1103, Japan; ^6^Division of Diabetes and Endocrinology, Department of Internal Medicine, Kobe University Graduate School of Medicine, Kobe 650-0017, Japan; ^7^The Integrated Center for Mass Spectrometry, Kobe University Graduate School of Medicine, Kobe 650-0017, Japan

## Abstract

The Zucker fatty (ZF) rat harboring a missense mutation (*fatty, fa*) in the leptin receptor gene (*Lepr*) develops obesity without diabetes; Zucker diabetic fatty (ZDF) rats derived from the ZF strain exhibit obesity with diabetes and are widely used for research on type 2 diabetes (T2D). Here we establish a novel diabetic strain derived from normoglycemic ZF rats. In our ZF rat colony, we incidentally found *fa/fa* homozygous male rats having reproductive ability, which is generally absent in these animals. During maintenance of this strain by mating *fa/fa* males and *fa/+* heterozygous females, we further identified *fa/fa* male rats exhibiting diabetes. We then performed selective breeding using the *fa/fa* male rats that exhibited relatively high blood glucose levels at 10 weeks of age, resulting in establishment of a diabetic strain that we designated Hos:ZFDM-*Lepr^fa^* (ZFDM). These *fa/fa* male rats developed diabetes as early as 10 weeks of age, reaching 100% incidence by 21 weeks of age, while none of the *fa/+* male rats developed diabetes. The phenotypic characteristics of this diabetic strain are distinct from those of normoglycemic ZF rats. ZFDM rat strain having high reproductive efficiency should serve as a more useful animal model of T2D.

## 1. Introduction

Type 2 diabetes (T2D) is a metabolic disorder characterized by high blood glucose due to insulin resistance and insulin deficiency. T2D accounts for more than 90% of all diabetes, and the prevalence of T2D and prediabetes is rapidly rising worldwide. Better treatment and prevention of T2D and its complications are needed to overcome the disease.

Animal models of T2D provide an opportunity to study the disease intensively. To date, many animal models of T2D have been established such as the Goto-Kakizaki (GK) rat [1], Otsuka Long-Evans Tokushima fatty (OLETF) rat [2], Spontaneously Diabetic Torii (SDT) rat [3], SDT fatty rat [4], Wistar fatty rat [5], Zucker diabetic fatty (ZDF) rat [6], *db/db* mouse [7], KK-*A* ^y^ mouse [8], Nagoya Shibata Yasuda (NSY) mouse [9], New Zealand obese (NZO) mouse [10], TallyHo (TH) mouse [11], and the Tsumura Suzuki obese diabetic (TSOD) mouse [12]. Among rat models of the disease, the ZDF rat is most widely used for study of T2D associated with obesity.

It is well recognized that a mutation in the leptin receptor gene (*Lepr*) causes infertility in the homozygous state. Thus, rats homozygous for a missense mutation (*fatty, fa*) in *Lepr* are infertile; these include the SDT fatty rat, WBN/Kob fatty rat [13], Wistar fatty rat, ZDF rat, and the Zucker fatty (ZF) rat [14], all of which harbor the *fa* mutation. The infertility of *fa/fa* homozygous animals requires maintenance and production of the strains by mating between *fa/*+ heterozygous animals. Only one male of eight pups obtained by this type of mating is expected to be *fa/fa* homozygous and serve as research material.

We establish here a novel diabetic rat strain, ZFDM, in which the *fa/fa* male rats have reproductive ability. We performed initial characterization of the strain to compare their phenotypic characteristics with those of the original ZF rats.

## 2. Materials and Methods

### 2.1. Animals

ZFDM rats (Hos:ZFDM-*Lepr* ^*fa*^,  *fa/fa* and *fa/*+) were provided by Hoshino Laboratory Animals, Inc. (Ibaraki, Japan). Normoglycemic ZF rats (Slc:Zucker, *fa/fa* and +/+) were purchased from Japan SLC, Inc. (Hamamatsu, Japan). All animals were maintained under specific pathogen free conditions at 23 ± 2°C and 55 ± 10% relative humidity with a 12 h light-dark cycle and were provided with water and a commercial diet CE-2 (CLEA Japan, Inc., Tokyo, Japan) at the Animal Facility of Kobe Biotechnology Research and Human Resource Development Center of Kobe University. All animal experiments were approved by the Committee on Animal Experimentation of Kobe University and carried out in accordance with the Guidelines for Animal Experimentation at Kobe University.

### 2.2. Phenotyping

The ZFDM and ZF rats were checked for body weight and nonfasting blood glucose level by a portable glucose meter (ANTSENSE III, HORIBA, Ltd., Kyoto, Japan) once a week from 5 to 21 weeks of age. Diabetes was defined as a blood glucose level equal to or higher than 300 mg/dl under *ad libitum* dietary conditions. At 6, 8, 12, 16, and 20 weeks of age, nonfasting whole blood samples were collected from the tarsal vein, and plasma samples were separated by centrifugation and stored at −80°C for later insulin measurement. In different batches, ZFDM rats at 8, 12, 16, 20, and 24 weeks of age were checked for body weight, body length (head to anus), and nonfasting blood glucose level and were sacrificed for histological analysis. Fasting whole blood samples were collected from the heart, and serum samples were separated by centrifugation and stored at −80°C for later leptin, adiponectin, and lipid parameter measurements. Body mass index (BMI) was calculated by dividing body weight (in grams) by body length (in centimeters) squared.

### 2.3. Assays

Plasma insulin levels were measured by insulin ELISA kit (Shibayagi Co., Ltd., Gunma, Japan). Serum leptin and adiponectin levels were measured by leptin ELISA kit (Morinaga Institute of Biological Science, Inc., Yokohama, Japan) and adiponectin ELISA kit (Otsuka Pharm. Co., Ltd., Tokyo, Japan), respectively. Serum lipid parameters were measured by Hitachi automatic analyzer 7070 (Hitachi High-Technologies Corporation, Tokyo, Japan). 

### 2.4. Histological Analysis

Histological analysis was performed using procedures essentially as described previously [15]. The pancreas was fixed in 10% neutral buffered formalin. The fixed specimens were embedded in paraffin, sectioned at 4 *μ*m, and stained with hematoxylin and eosin for histopathological examination.

### 2.5. Statistical Analysis

Data are expressed as means ± SEM. Differences in blood glucose level, body weight, body length, BMI, plasma insulin level, serum leptin level, serum adiponectin level, and serum lipid parameters were assessed using Welch's *t*-tests. Differences for which the *P* value was <0.05 were regarded as statistically significant.

## 3. Results

### 3.1. Establishment of a Normoglycemic ZF Rat Colony Having High Reproductive Efficiency

**Figure 1:**
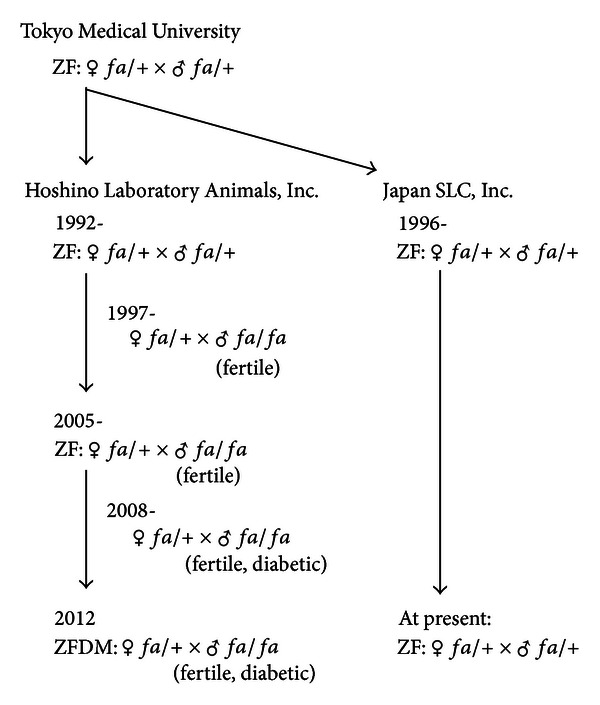
Establishment of a diabetic rat strain ZFDM from normoglycemic ZF strain.

To improve the reproductive efficiency of our ZF rat colony, we have been trying to obtain *fa/fa* animals having reproductive ability since 1992 (Figure 1). In 1997, we fortunately identified *fa/fa* males having reproductive ability and tried to maintain the colony by using these *fa/fa* males. In 2005, we successfully established an outbred ZF rat colony in which *fa/fa* males having reproductive ability were frequently and continuously obtained. We have been maintaining this colony by mating between *fa/*+ females and 10- to 15-week-old *fa/fa* males.

### 3.2. Establishment of a Diabetic Rat Colony, Hos:ZFDM-*Lepr* ^*fa*^

In 2008, during maintenance of the above-mentioned ZF rat colony, we incidentally identified one 25-week-old *fa/fa* male rat exhibiting diabetes (Figure 1). Afterward, we measured blood glucose levels of this *fa/fa* male's descendants at 10 weeks of age and selected the animals showing relatively high blood glucose levels to maintain the colony. Consecutive maintenance by this type of selective breeding for more than 10 generations resulted in establishment of an outbred diabetic rat colony, which we designated Hos:ZFDM-*Lepr* ^*fa*^ (ZFDM). It is most noteworthy that the *fa/fa* males in this ZFDM rat colony are fertile and diabetic.

### 3.3. Phenotypic Characterization of the ZFDM Strain

**Figure 2:**
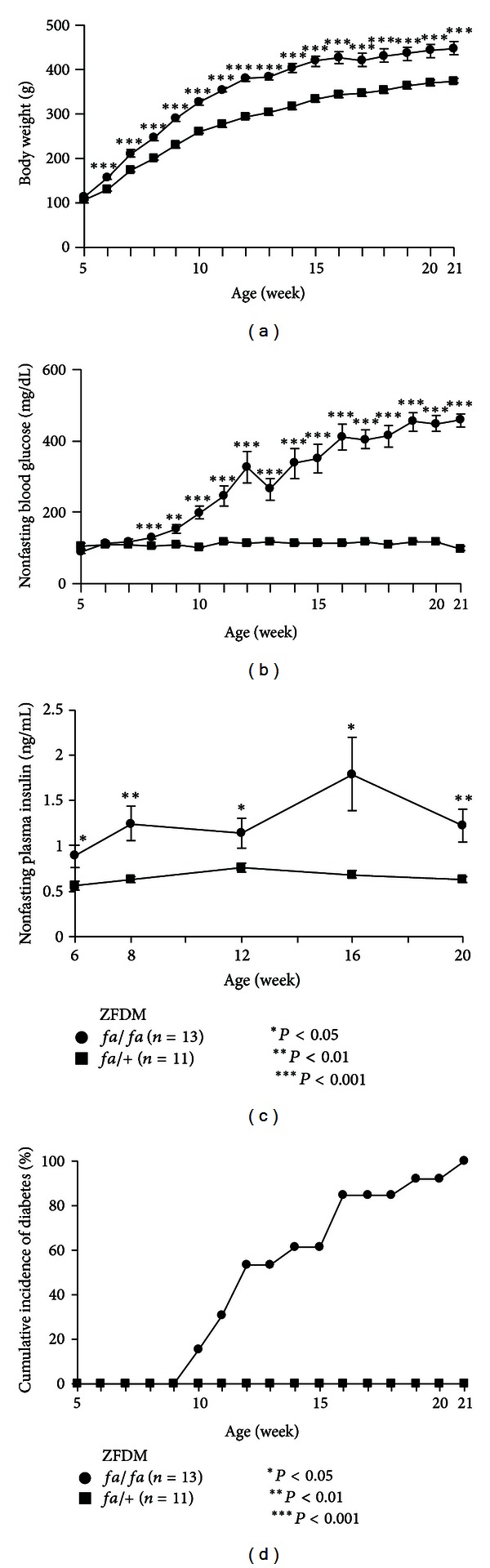
Longitudinal phenotypic characterization of the ZFDM strain. (a) Body weight, (b) nonfasting blood glucose level, (c) nonfasting plasma insulin level, and (d) cumulative incidence of diabetes of *fa/fa* (*n* = 13) and *fa/*+ (*n* = 11) male rats in the ZFDM strain. Welch's *t*-test was used for comparisons between *fa*/*fa* and *fa/*+ rats.

We then compared body weight and nonfasting blood glucose level of *fa/fa* and *fa/*+ male rats in the ZFDM strain from 5 to 21 weeks of age (Figures 2(a) and 2(b)). Differences in body weight between *fa/fa* and *fa/*+ rats were evident as early as 6 weeks of age (*fa/fa *158.4 ± 4.5 g versus *fa*/+130.6 ± 4.8  g, *P* = 0.0004) and were increased gradually until 21 weeks of age (*fa/fa *448.5 ± 15.3 g versus *fa*/+  375.3 ± 5.8 g, *P* = 0.0004). Differences in nonfasting blood glucose level between *fa/fa* and *fa*/+ rats were evident as early as 8 weeks of age (*fa/fa *128.2 ± 4.7 mg/dL versus *fa*/+  104.7 ± 2.1 mg/dL,  *P* = 0.0003) and were increased markedly until 21 weeks of age (*fa/fa *464.2 ± 30.0 mg/dL versus *fa*/+  118.6 ± 2.1 mg/dL, *P* < 0.0001). Nonfasting plasma insulin level was significantly higher in *fa/fa* rats than that in *fa*/+ rats at 6 weeks of age (*fa/fa *0.89 ± 0.12 ng/mL versus *fa*/+  0.56 ± 0.04 ng/mL, *P* = 0.026), and that in *fa/fa* rats was increased until 16 weeks of age and then decreased but remained significantly higher than that of *fa*/+ rats at 20 weeks of age (*fa/fa *1.23 ± 0.18 ng/mL versus *fa*/+  0.62 ± 0.03 ng/mL, *P* = 0.006) (Figure 2(c)). The *fa/fa* rats developed diabetes (nonfasting blood glucose levels ≥ 300 mg/dL) as early as 10 weeks of age and the cumulative incidence of diabetes reached 100% by 21 weeks of age (Figure 2(d)). In contrast, none of the *fa*/+ rats developed diabetes.

**Figure 3:**
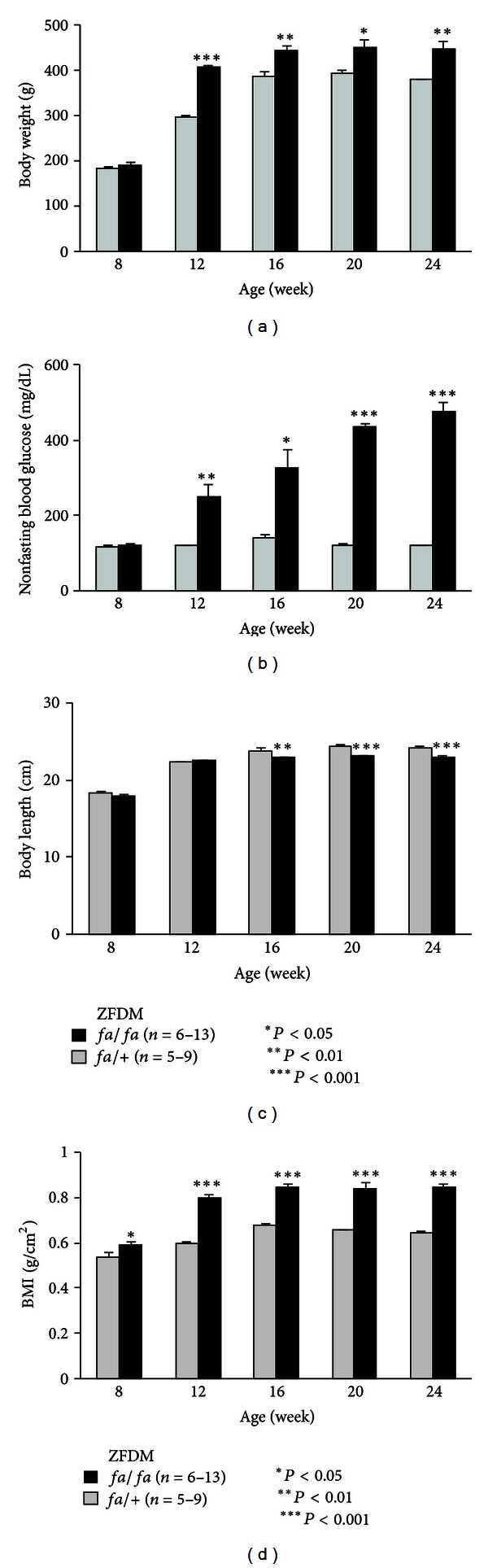
Cross-sectional phenotypic characterization of the ZFDM strain. (a) Body weight, (b) nonfasting blood glucose level, (c) Body length, and (d) BMI of *fa/fa* (*n* = 6, 6, 6, 6, and 13 for 8, 12, 16, 20, and 24 weeks of age, resp.) and *fa/*+ (*n* = 6, 6, 5, 5, and 9 for 8, 12, 16, 20, and 24 weeks of age, resp.) male rats in the ZFDM strain. Welch's *t* test was used for comparisons between *fa/fa* and *fa/*+ rats.

**Figure 4:**
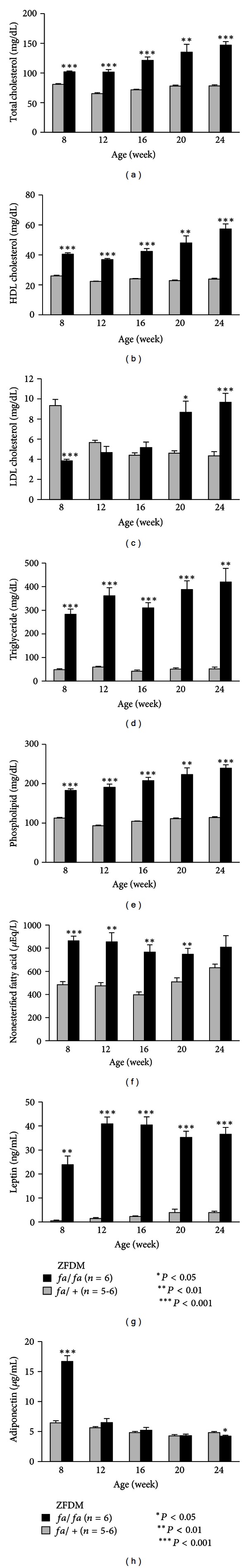
Serum lipid parameters and adipokines of the ZFDM strain. Fasting serum levels of (a) total cholesterol, (b) HDL cholesterol, (c) LDL cholesterol, (d) triglyceride, (e) phospholipid, (f) nonesterified fatty acid, (g) leptin, and (h) adiponectin of *fa/fa* (*n* = 6) and *fa/*+ (*n* = 6, 6, 5, 5, and 6 for 8, 12, 16, 20, and 24 weeks of age, resp.) male rats in the ZFDM strain. Welch's *t* test was used for comparisons between *fa/fa* and *fa/*+ rats.

In different batches of the experiment, we compared body weight, nonfasting blood glucose level, body length, and BMI of *fa/fa* and *fa*/+ male rats in the ZFDM strain at 8, 12, 16, 20, and 24 weeks of age (Figure 3). Body weight and nonfasting blood glucose level of both animals showed similar tendencies to those described above (Figures 3(a) and 3(b)). Body length was significantly larger in *fa/*+ rats than that in *fa/fa* rats at 16 weeks of age and later (16 weeks of age: *fa/fa *22.9 ± 0.2 cm versus *fa*/+  23.9 ± 0.2 cm, *P* = 0.007) (Figure 3(c)). Differences in BMI between *fa/fa* and *fa/*+ rats were evident at 8 weeks of age (*fa/fa *0.59 ± 0.01 g/cm^2^ versus *fa*/+  0.54 ± 0.01 g/cm^2^, *P* = 0.028), were increased markedly by 12 weeks of age, and were maintained until 24 weeks of age (*fa/fa *0.84 ± 0.02 g/cm^2^ versus *fa*/+  0.65 ± 0.01 g/cm^2^, *P* < 0.0001) (Figure 3(d)). Levels of serum lipid parameters and adipokines are shown in Figure 4. Most of the lipid parameters and leptin levels were higher in *fa/fa* rats than those in *fa/*+ rats. In contrast, the adiponectin level in *fa/fa* rats was decreased with age and was lower than that in *fa/*+ rats at 24 weeks of age (Figure 4(h)).

### 3.4. Comparison of the Characteristic Features of the ZFDM Strain with Those of the Normoglycemic ZF Strain

**Figure 5:**
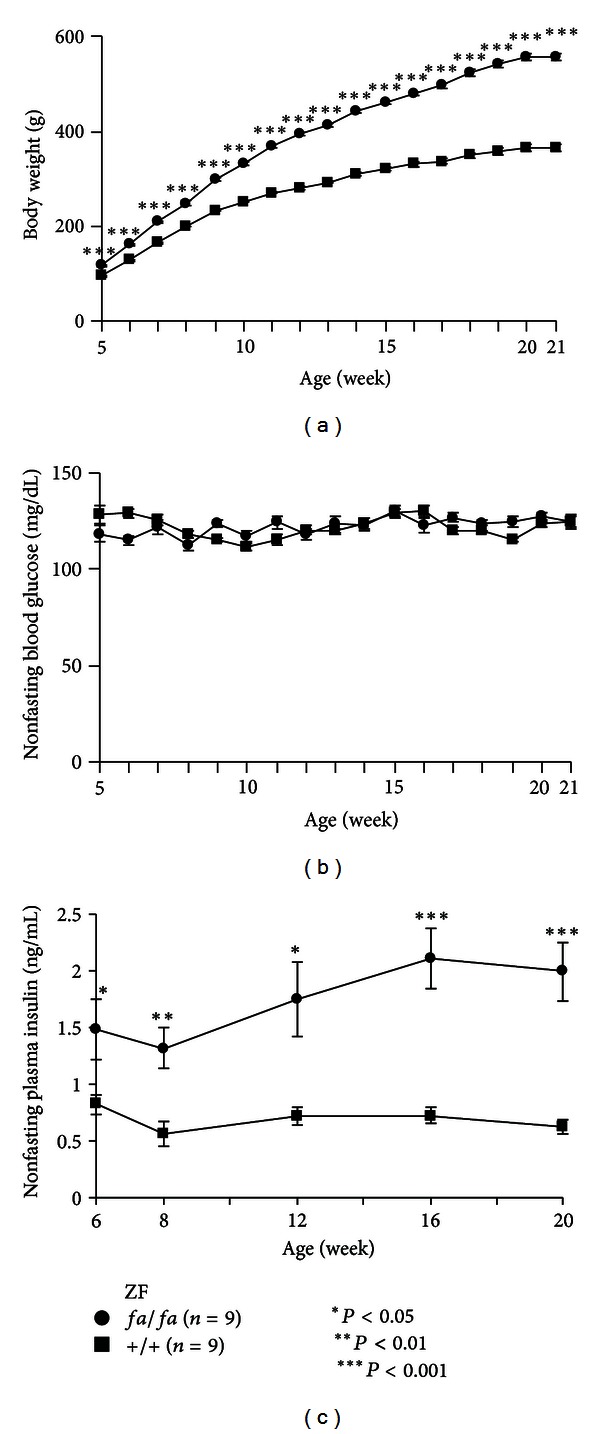
Longitudinal phenotypic characterization of the ZF strain. (a) Body weight, (b) nonfasting blood glucose level, and (c) nonfasting plasma insulin level of *fa/fa* (*n* = 9) and +/+ (*n* = 9) male rats in the ZF strain. Welch's *t* test was used for comparisons between *fa/fa* and +/+ rats.

To compare the phenotype of the ZFDM strain and that of the normoglycemic ZF strain, we measured body weight and nonfasting blood glucose level of *fa/fa* and +/+ rats in the ZF strain from 5 to 21 weeks of age (Figures 5(a) and 5(b)). Differences in body weight between *fa/fa* and +/+ rats in the ZF strain were evident as early as 5 weeks of age (data at 6 weeks of age for comparison with the ZFDM strain: *fa/fa *161.8 ± 3.0 g versus +/+  129.2 ± 2.3 g, *P* < 0.0001) and were gradually increased until 21 weeks of age (*fa/fa *558.8 ± 7.4 g versus +/+  367.0 ± 6.9 g, *P* < 0.0001) (Figure 5(a)). While the body weights of *fa/fa* rats in both ZFDM and ZF strains were similar at earlier ages, those of *fa/fa* rats in the ZFDM strain were significantly lower at 11 weeks of age and later, most likely due to the onset of diabetes (Supplementary Figure 1(a) available online at http://dx.doi.org/10.1155/2013/103731). There was no difference in nonfasting blood glucose level between *fa/fa* and +/+ rats in the ZF strain during the experimental period, and none of the *fa/fa* and +/+ rats in the ZF strain exhibited diabetes by 21 weeks of age (Figure 5(b)); the character is different from that of the ZFDM strain (Supplementary Figure 1(b)). Nonfasting plasma insulin level was significantly higher in *fa/fa* rats than that in +/+ rats at 6 weeks of age (*fa/fa *1.49 ± 0.27 ng/mL versus +/+  0.83 ± 0.09 ng/mL, *P* = 0.045) and that in *fa/fa *rats was increased until 20 weeks of age (*fa/fa *2.00 ± 0.26 ng/mL versus +/+  0.62 ± 0.06 ng/mL, *P* = 0.0007) (Figure 5(c)).

### 3.5. Histological Characterization of the Pancreas of the ZFDM Strain

**Figure 6:**
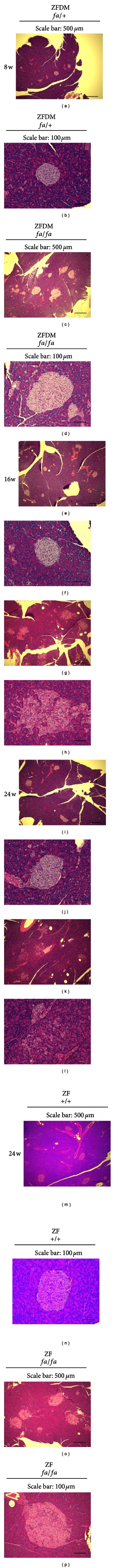
Histological characterization of the pancreas of the ZFDM and ZF strains. Representative pancreas histology of *fa/*+ (a, b, e, f, i, and j) and *fa/fa* (c, d, g, h, k, and l) male rats in the ZFDM strain and +/+ (m and n) and *fa/fa* (o and p) male rats in the ZF strain. Hematoxylin and eosin staining.

While there were no obvious pathological changes in endocrine and exocrine pancreas of *fa/*+ rats in the ZFDM strain (Figures 6(a), 6(b), 6(e), 6(f), 6(i), and 6(j)), various degrees of pathological change were observed in the pancreas of the *fa/fa* rats at 16 and 24 weeks of age, including loss of islet architecture and fibrosis (Figures 6(g), 6(h), 6(k), and 6(l)). To determine whether these pathological changes are specific to the *fa/fa* rats in the ZFDM strain, we performed histological characterization of the pancreas in the normoglycemic ZF strain. Although the pancreatic islets of *fa/fa* rats in the ZF strain were enlarged, islet architecture was maintained even at 24 weeks of age (Figures 6(o) and 6(p)). Similarly to the *fa/*+ rats in the ZFDM strain, +/+ rats in the ZF strain exhibited no obvious pathological changes in the pancreas (Figures 6(m) and 6(n)). 

## 4. Discussion

In this study, we describe the establishment and initial characterization of a novel type 2 diabetes model derived from normoglycemic ZF rats. We first established an outbred ZF rat colony in which *fa/fa* males have reproductive ability. We then incidentally identified an *fa/fa* male rat exhibiting diabetes and performed selective breeding using *fa/fa* male rats showing relatively high blood glucose levels at 10 weeks of age. We successfully maintained this colony by mating between *fa/fa* male and *fa/*+ female rats, which resulted in establishment of an outbred diabetic rat colony that we designated Hos:ZFDM-*Lepr* ^*fa*^. The male *fa/fa* rats in the ZFDM strain are fertile, exhibit obesity, and develop diabetes as early as 10 weeks of age, reaching 100% incidence at around 20 weeks of age.

The ZDF strain was established in 1990 [6] and has been widely used to study T2D associated with obesity. The ZDF strain is an inbred rat model of early-onset diabetes in which all of the *fa/fa* male rats develop diabetes at 10 to 12 weeks of age when fed a special diet of Purina 5008 (Charles River Laboratories International, Inc., Wilmington, MA, USA). The phenotype is homogeneous, mainly due to the facts that the strain is genetically inbred and the special diet is provided. In contrast, the ZFDM strain is an outbred rat model of young- to middle-aged adult-onset diabetes in which the *fa/fa* male rats develop diabetes as early as 10 weeks of age and most develop diabetes at 12 to 20 weeks of age (Table 1). After the onset of diabetes, high blood glucose levels are maintained, a feature is similar to that of the ZDF strain. Since the prediabetic stage of the ZFDM strain is much longer than that of the ZDF strain, the ZFDM strain is more suitable for pathophysiological studies of diabetes and therapeutic intervention studies. In the ZDF strain, the *fa/fa* male rats exhibit diabetic complications including nephropathy [16] and neuropathy [17]. Given that the ZFDM strain harbors a similar genetic background to that of the ZDF strain and exhibits severe diabetes, diabetic complications found in the ZDF strain may well occur in the ZFDM strain.

Usually, humans, mice, and rats lacking normal *Lepr* are infertile due to hypogonadotropic hypogonadism. However, there is some evidence for spontaneous pubertal development in humans homozygous for a splicing mutation resulting in LEPR lacking both the transmembrane and intracellular domains, among whom one woman was able to give birth to a healthy son [18, 19]. In addition, human subjects with *LEPR* missense mutations have been reported to show less severe clinical features than those with the splicing mutation described above [20]. In rat species, L. M. Zucker and T. F. Zucker reported that *fa/fa* female rats are always infertile [14], while Chelich and Edmonds found that a small portion of young *fa/fa* female rats are fertile [21]. Furthermore, young *fa/fa* male rats are more likely to be fertile; about 70% of *fa/fa* male rats were reported to be fertile in a colony at Vassar College [22], but their current status is unknown. In our ZF rat colony, more than 10 generations of selective breeding for fertility of *fa/fa* male rats enabled us to obtain fertile *fa/fa* male rats stably.

## 5. Conclusions

We have established a novel diabetic rat strain, ZFDM, in which *fa/fa* male rats are fertile. In this strain, *fa/fa* male rats develop diabetes as early as 10 weeks of age, which reaches 100% incidence at around 20 weeks of age, while no *fa/*+ rats develop diabetes. The ZFDM rat strain possesses high reproductive efficiency and therefore should serve as a useful model of young- to middle-aged adult-onset T2D in studies of the pathophysiology, therapeutic interventions, and complications of the disease. 

## Supplementary Material

Supplemental Figure 1: Comparison of phenotypes between *fa/fa* rats in the ZFDM and ZF strains. (a) body weight and (b) non-fasting blood glucose level of ZFDM (n=13) and ZF (n=9) male rats. Welch's t test was used for comparisons between ZFDM and ZF strains.

## Figures and Tables

**Table 1:** Characteristics of the ZDF and ZFDM strains.

	ZDF	ZFDM
Origin	ZF colony at Eli Lilly Research Laboratories (Indianapolis, IN)	ZF colony at Tokyo Medical University (Tokyo, Japan)
Genetic control	Inbred	Outbred
Obesity*	Early onset (6–10 wk)	Early onset (6–12 wk)
Diabetes*	Young-age onset (8–12 wk)	Young- to middle-age onset (10–20 wk)
Special diet	Purina 5008	Not needed
Breeding	♀ *fa*/+ × ♂ *fa*/+	♀ *fa*/+ × ♂ *fa*/*fa*

*Characteristics of *fa*/*fa* male rats in the ZDF and ZFDM strains.
