# Supplementary material for: A Novel Rat Model of Type 2 Diabetes: The Zucker Fatty Diabetes Mellitus ZFDM Rat
Source: J Diabetes Res. 2013 Feb 26;2013:103731. doi: 10.1155/2013/103731 (PMC3647587; doi:10.1155/2013/103731)
Supplement: Supplementary file 1 — Supplemental Figure 1: Comparison of phenotypes between fa/fa rats in the ZFDM and ZF strains. (a) body weight and (b) non-fasting blood glucose level of ZFDM (n=13) and ZF (n=9) male rats. Welch's t test was used for comparisons between ZFDM and ZF strains. [file 103731.f1.pdf]

Supplementary Figure 1

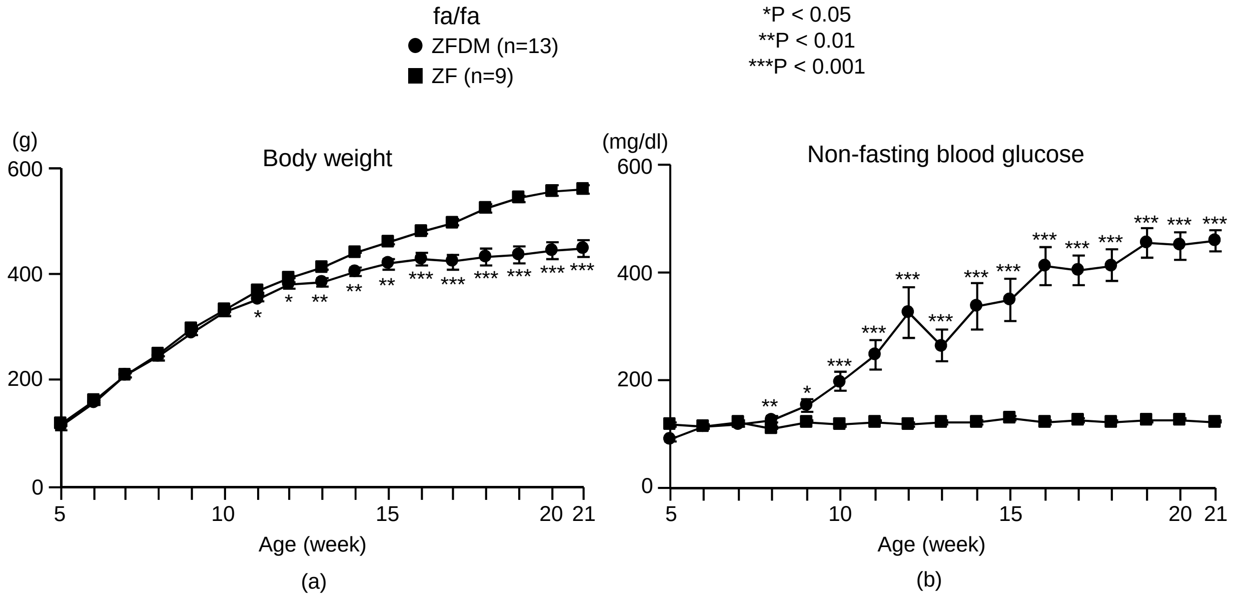

Supplementary figure 1:
Comparison of phenotypes between *fa/fa* rats in the ZFDM and ZF strains.
(a) body weight and (b) non-fasting blood glucose level of ZFDM (n=13) and ZF (n=9) male rats.
Welch's t test was used for comparisons between ZFDM and ZF strains.
